# Outcome of Using Intraventricular Plus Intravenous Polymyxin B in Post-neurosurgical Patients With Multi/Extensively Drug-Resistant Gram-Negative Bacteria-Induced Intracranial Infection

**DOI:** 10.3389/fmed.2022.913364

**Published:** 2022-07-06

**Authors:** Hangyang Li, Wenqiao Yu, Guobin Wang, Hongliu Cai

**Affiliations:** Department of Critical Care Medicine, First Affiliated Hospital, Zhejiang University School of Medicine, Hangzhou, China

**Keywords:** intraventricular polymyxin B, colistin, neurosurgery, central nervous system (CNS) infection, multidrug-resistant (MDR)/extensively drug-resistant (XDR) bacteria

## Abstract

**Introduction:**

Post-neurosurgical central nervous system (CNS) infection caused by multidrug-resistant (MDR)/extensively drug-resistant (XDR) Gram-negative bacteria remains a major clinical challenge. This study describes our experience of treating such patients with combined intraventricular (IVT) and intravenous (IV) polymyxin B administration.

**Methods:**

This retrospective study included six patients with post-neurosurgical CNS infections of carbapenem-resistant *Acinetobacter baumannii* (CRAB) or carbapenem-resistant *Klebsiella pneumoniae* (CRKP). All patients were treated in the intensive care unit (ICU) of First Affiliated Hospital, Zhejiang University School of Medicine (Hangzhou, China) between November 2020 and November 2021, and all received IVT plus IV polymyxin B. Data including patients' characteristics, therapeutic process, symptoms, cerebrospinal fluid (CSF) examination, laboratory tests, and complications were collected.

**Results:**

Six patients with post-neurosurgical CNS infection were enrolled in the study. The patients comprised five males and one female, and the average age was 58 years (range, 38–73 years). Four out of the six cases were CRAB-positive in CSF culture, while two cases were CRKP-positive. The mean duration of polymyxin B administration was 14 ± 5.69 days (range, 6–20 days). The average period of patients reaching CSF sterilization was 10.33 ± 3.67 days (range, 5–14 days). All six cases were cured without acute kidney injury or epilepsy.

**Conclusion:**

IVT plus IV polymyxin B is a safe and effective treatment for post-neurosurgical patients with intracranial infection caused by MDR/XDR Gram-negative bacteria.

## Introduction

Central nervous system (CNS) infection is one of the most common complications following neurosurgery and can be difficult to treat due to the blood–brain barrier (BBB). The BBB is crucial for the protection of the CNS against microbial entry from the circulation, but can also prevent various drugs from entering the brain.

The microbes responsible for CNS infection are predominantly Gram-negative and Gram-positive bacteria. According to the 2021 China Antimicrobial Surveillance Network (CHINET) report,^1^ the detection rate of Gram-negative bacteria *Acinetobacter baumannii* (AB) and *Klebsiella pneumoniae* (KP) in the CNS from patients with intracranial infection was 11.3 and 9.1%, respectively. Moreover, multidrug-resistant (MDR)/extensively drug-resistant (XDR) bacterial strains in critical-care patients have become much more common in recent years, making the treatment of CNS infections increasingly difficult. Furthermore, meningitis-related mortality is significantly associated with carbapenem resistance (1). Fortunately, the MDR/XDR pathogens remain susceptible to the polymyxins, although only a small proportion of the intravenous (IV) polymyxin dosage reaches the CNS infection site due to limited penetration of the BBB by this class of antibiotics (2, 3). Consequently, the cerebrospinal fluid (CSF) distribution of polymyxin is very low.

According to the 2017 guidelines of the American Society for Infectious Diseases (IDSA) (4), intraventricular (IVT) injection combined with IV polymyxin B or colistin is recommended for the treatment of intracranial MDR Gram-negative bacterial infections. Multiple studies have suggested IVT+IV colistin or polymyxin B is the optimal treatment option for CNS infections with MDR/XDR Gram-negative bacteria (5–7). Current IDSA guidelines for IVT polymyxin B recommend a dose of 5 mg once daily for 10–21 days (4). However, there are limited data on the safety, efficacy, and optimal dose/duration of polymyxin B for patients with intracranial infection. The present study describes our experience of six patients with post-neurosurgical CNS infections of carbapenem-resistant *Acinetobacter baumannii* (CRAB) or carbapenem-resistant *Klebsiella pneumoniae* (CRKP) and their treatment with IVT+IV polymyxin B.

## Methods

In this retrospective case series, the medical records of six patients with post-neurosurgical CNS infections of CRAB/CRKP, who were hospitalized in the intensive care unit (ICU) of First Affiliated Hospital, Zhejiang University School of Medicine (Hangzhou, China) between November 2020 and November 2021, were reviewed.

### Inclusion and Exclusion Criteria

Inclusion criteria for enrollment in the study included: (1) patients ≥18 years old; (2) cases with both clinical and imaging evidence of post-neurosurgical CNS infection; (3) laboratory tests (routine and biochemical tests) of CSF suggestive of CNS infection; (4) detection of CRAB/CRKP in CSF culture; (5) concomitant use of IVT and IV polymyxin B.

Exclusion criteria for enrollment in the study were: (1) history of intracranial infection before craniotomy; (2) previous treatment with IV polymyxin B/colistin for intracranial infection or infection at other positions in the past 3 months; (3) administration of either IV polymyxin B or IVT polymyxin B alone.

### Microbiological Methods

The broth microdilution method was used for bacterial strain identification and antimicrobial susceptibility tests. One exception was the method for ceftazidime-avibactam susceptibility, which was tested by the Kirby-Bauer method. Antimicrobial susceptibility results are presented in Table 1.

**Table 1:** Antibiotic minimum inhibitory concentration (MIC, μg/mL)/susceptibility of CSF isolates from patients enrolled in the study.

**Antibiotic**	**Case (CSF isolate)**					
	**1 (AB)**	**2 (AB)**	**3 (AB)**	**4 (AB)**	**5 (KP)**	**6 (KP)**
Polymyxin	≤ 0.5/S	≤ 0.5/S	≤ 0.5/S	≤ 0.5/S	1/S	1/S
Tigecycline	4/I	4/I	4/I	2/S	1/S	0.5/S
*Ceftazidime-Avibactam	NT	NT	NT	NT	27 mm/S	25 mm/S
Meropenem	≥16/R	≥16/R	≥16/R	≥16/R	≥16/R	NT
Imipenem	≥16/R	≥16/R	≥16/R	≥16/R	≥16/R	≥16/R
Piperacillin-tazobactam	≥128/R	≥128/R	≥128/R	≥128/R	≥128/R	≥128/R
Cefoperazone-sulbactam	≥64/R	≥64/R	32/R	≥64/R	≥64/R	≥64/R
Ticarcillin-clavulanic acid	≥128/R	≥128/R	≥128/R	≥128/R	≥128/R	NT
Ceftazidime	≥64/R	≥64/R	≥64/R	≥64/R	≥64/R	≥64/R
Cefepime	≥32/R	≥32/R	≥32/R	≥32/R	≥32/R	≥32/R
Amikacin	4/R	8/R	≤ 2/S	NT	≥64/R	≥64/R
Tobramycin	≥16/R	≥16/R	≤ 1/S	≤ 1/S	≥16/R	NT
Ciprofloxacin	≥4/R	≥4/R	≥4/R	≥4/R	≥4/R	NT
Levofloxacin	≥8/R	≥8/R	≥8/R	≥8/R	≥8/R	≥8/R
Doxycycline	≥16/R	≥16/R	1/S	≥16/R	≥16/R	NT
Minocycline	8/I	8/I	≤ 1/S	≥16/R	≥16/R	NT
Sulfamethoxazole-trimethoprim	≤ 20/S	≥320/R	≥320/R	≥320/R	≥320/R	≥320/R

*The broth microdilution method was used for all antibiotics except Ceftazidime-Avibactam, which was tested by the Kirby-Bauer method.

*CSF, cerebrospinal fluid; AB, Acinetobacter baumannii; KP, Klebsiella pneumoniae; S, susceptible; I, intermediate; R, resistant; NT, not tested*.

### Treatments

After diagnosis of a CNS infection, the external ventricular drain (EVD) was removed and replaced. Before the detection of CRAB/CRKP was confirmed, systemic control of infection with intravenous antibiotics—including meropenem, vancomycin, cefoperazone-sulbactam, and piperacillin-tazobactam—was performed based on clinical experience. Polymyxin B administration was initiated once positive culture of CRAB/CRKP was confirmed in the CSF. IVT polymyxin B at a dose of 5 mg (50,000 units) per 24 h, plus IV polymyxin B at a dose of 1.5 mg/kg every 12 h was given (4, 8). IVT polymyxin B was diluted in 5 mL of 0.9% saline solution and injected into the ventricle through the EVD. Thereafter, the EVD was flushed with 2 mL of 0.9% saline solution and clamped for 2 h. After 3 days of treatment, CSF samples were collected from the EVD every 2 days.

### Clinical Outcomes

Clinical cure was defined as: (1) remission of the clinical symptoms and signs of CNS infection; (2) routine and biochemical tests of CSF back to normal; (3) three consecutive negative CSF cultures.

### Data Collection

For each case, collected data included clinical characteristics (age, gender, primary disease, surgeries, foreign body), therapeutic process (antibiotic usage prior to positive CSF culture, concomitant infection, time from symptoms onset to IVT polymyxin B administration, IVT+IV polymyxin B duration, CSF sterilization time), symptoms and signs [temperature, Glasgow Coma Scale (GCS)], CSF examination (culture, susceptibility, polymorphonuclear leukocytes, glucose, protein), laboratory tests (white blood cell (WBC), C-reactive protein (CRP), procalcitonin (PCT), creatinine, glucose), and complications [hydrocephalus, additional surgery of ventriculoperitoneal shunt (VPS) for hydrocephalus, epilepsy, acute kidney injury (AKI), and skin hyperpigmentation]. Adverse events related to polymyxin B were graded using the Common Toxicity Criteria for Adverse Events (CTCAE), version 5.0.

### Data Analysis

Statistical analysis was performed using RStudio (version 2021.09.1+372). Numeric data with a normal distribution were shown as mean ± standard deviation, and a paired *t*-test was conducted for the comparison between pre- and post-treatment variables. Non-normally distributed data were presented as median with interquartile range, and statistical analysis was performed by the Wilcoxon test. *P* < 0.05 was considered statistically significant.

## Results

Between November 2020 and November 2021, six patients with post-neurosurgical CNS infection treated with IVT+ IV polymyxin B were enrolled in the study, including five males and one female. The age of the patients ranged from 38 to 73 years with an average of 58 years. Primary neurological diseases and surgical methods are shown in Table 2. An EVD catheter was placed for all patients during the operation.

**Table 2:** Clinical characteristics of patients enrolled in the study.

**Case**	**Age (years)**	**Gender**	**Primary disease**	**Surgeries**	**Foreign body**
1	38	Male	Aneurysm	Aneurysm embolization + Hematoma evacuation + EVD	EVD
2	64	Male	Moyamoya disease	Hematoma evacuation + EVD	EVD
3	61	Male	TBI	Hematoma evacuation + EVD	EVD
4	73	Female	TBI	Hematoma evacuation + EVD	EVD
5	57	Male	ICH	Hematoma evacuation + EVD	EVD
6	55	Male	Cerebellar infarction	Decompressive craniectomy + EVD	EVD

*TBI, traumatic brain injury; ICH, intracerebral hemorrhage; EVD, external ventricular drain*.

Of the six cases, four patients were confirmed with CRAB infection in CSF culture, three of whom also showed CRAB in sputum samples. The other two cases were CRKP-positive in both CSF and sputum culture. Before CSF culture results were available, all patients were treated with meropenem and vancomycin (Table 3). Additionally, three patients received cefoperazone-sulbactam and one received piperacillin-tazobactam. The time between onset of symptoms and known bacterial susceptibility was 3 days, then the treatment of IVT+IV polymyxin B was administered based on drug susceptibility tests. The mean duration of IVT+IV polymyxin TB administration was 14 ± 5.69 days (range, 6–20 days), while the mean duration to achieve sterile CSF was 10.33 ± 3.67 days (range, 5–14 days) (Table 3).

**Table 3:** Cerebrospinal fluid (CSF) culture and treatment strategies for each patient.

**Case**	**CSF isolate**	**Susceptibility**	**Antibiotics usage prior to positive CSF culture**	**Concomitant infection**	**Time from onset of symptoms to IVT+IV polymyxin B administration (days)**	**Dose of IVT polymyxin B (mg/day)**	**Dose of IV polymyxin B (mg/kg/12 h)**	**Duration of IVT+IV polymyxin B (days)**	**CSF sterilization time (days)**
1	AB	Carbapenem-resistant; Susceptible to polymyxin	Cefoperazone-sulbactam; Meropenem; Vancomycin	Pneumonia (AB+PA)	5	5	1.5	15	13
2	AB	Carbapenem-resistant; Susceptible to polymyxin	Meropenem; Vancomycin	No	3	5	1.5	9	7
3	AB	Carbapenem-resistant; Susceptible to polymyxin	Cefoperazone-sulbactam; Meropenem; Vancomycin	Pneumonia (AB)	5	5	1.5	6	5
4	AB	Carbapenem-resistant; Susceptible to polymyxin	Piperacillin-tazobactam; Meropenem; Vancomycin	Pneumonia (AB)	7	5	1.5	20	13
5	KP	Carbapenem-resistant; Susceptible to polymyxin	Cefoperazone-sulbactam; Meropenem; Vancomycin	Pneumonia (KP)	6	5	1.5	14	10
6	KP	Carbapenem-resistant; Susceptible to polymyxin	Meropenem; Vancomycin	Pneumonia (KP)	4	5	1.5	20	14

*CSF, cerebrospinal fluid; AB, Acinetobacter baumannii; KP, Klebsiella pneumoniae; PA, Pseudomonas aeruginosa; IVT, intraventricular; IV, intravenous*.

Clinical features and laboratory data before and after treatment are presented in Table 4. The median CSF/blood glucose ratio of the six patients increased significantly following IVT plus IV polymyxin B administration (0.013 pre-treatment vs. 0.684 post-treatment, *P* < 0.05). Glucose in CSF (0.1 vs. 4.95 mmol/L, *P* < 0.05) and GCS score (5.00 ± 2.28 vs. 7.17 ± 3.43, *P* < 0.05) also improved markedly after the polymyxin B treatment. Meanwhile, the number of polymorphonuclear leukocytes in CSF showed a significant decrease compared with pre-treatment tests (11,525 vs. 218 cells/μL, *P* < 0.05). Serum creatinine was not significantly different from the baseline level (63.00 ± 38.13 vs. 58.50 ± 28.02 μmol/L, *P* = 0.45).

**Table 4:** Clinical symptoms and laboratory data in patients.

**Laboratory measurement**	**Beginning of CNS infection**	**Resolution of CNS infection**	***P*-value**
Temperature (°C)	39.3 ± 0.45	37.13 ± 0.21	<0.001
GCS	5.00 ± 2.28	7.17 ± 3.43	0.027
**Serum**
WBC count (×10^9^/L)	18.85 ± 6.41	7.99 ± 2.21	0.004
CRP (mg/L)	152.03 ± 48.96	19.23 ± 11.92	0.002
PCT (ng/mL)	0.86 ± 0.55	0.21 ± 0.20	0.023
Creatinine (μmoI/L)	63.00 ± 38.13	58.50 ± 28.02	0.45
**CSF**
Leukocytes (cells/μL)	11,525 (5,275, 21,412.5)	218 (31, 391.5)	0.028
Glucose (mmol/L)	0.1 (0.1, 0.7)	4.95 (4.0, 5.9)	0.028
CSF/blood glucose	0.013 (0.0, 0.1)	0.684 (0.7, 0.7)	0.028
Protein (g/L)	5.96 (3.4, 8.0)	2.41 (1.5, 4.2)	0.116

*CNS, central nervous system; GCS, Glasgow Coma Scale; WBC, white blood cell; CRP, C-reactive protein; PCT, procalcitonin; CSF, cerebrospinal fluid. Data are presented as the mean ± standard deviation or median (interquartile range)*.

Clinical cure was achieved in all patients (100%). During treatment and follow-up, three patients (50%) presented with hydrocephalus because of severe infection and two of them subsequently underwent VPS surgery for this complication. Four patients (66.6%) had skin hyperpigmentation after polymyxin usage. No drug-related epilepsy or AKI were found (Table 5). Furthermore, no adverse events of grade 3 or higher were observed.

**Table 5:** Clinical outcomes and complications.

**Outcome/complication**	**No. cases (%)**	**No. adverse events (%)**	
		**Grade 1–2**	**Grade 3 or higher**
Cured case	6 (100)	–	–
28-day mortality	0 (0)	–	–
**Adverse events**
Epilepsy	0 (0)	0 (0)	0 (0)
Acute kidney injury (AKI)	0 (0)	0 (0)	0 (0)
Skin hyperpigmentation	4 (66.6)	4 (66.6)	0 (0)

## Discussion

CHINET (see footnote 1) online data for 2021 show that the isolation rates of AB and KP in CSF were 11.3 and 9.1%, respectively, ranking second and third. Furthermore, a marked increase in drug resistance of AB to imipenem was seen from 31% in 2005 to 71.5% in 2021, and a similar increase was seen in the resistance rate of AB to meropenem—from 39% in 2005 to 72.3% in 2021. Increased resistance of KP to these two antibiotics was also observed (from 3.0% in 2005 to 23.1% in 2021 for imipenem, and from 2.9% in 2005 to 24.4% in 2021 for meropenem). Clinical data on CRAB/CRKP in our hospital exhibited similar trends.

CNS infection is one of the most common complications after neurosurgery (9), and such CNS infections caused by MDR/XDR Gram-negative bacteria after neurosurgery have a higher mortality rate. Treatment of these infections is difficult due to the limited choice of antibiotics and the presence of the BBB (10). Polymyxins are one of the most effective classes of antibiotics against MDR/XDR Gram-negative bacteria (11). However, polymyxins have difficulty penetrating through the BBB when administered by the IV route due to the polycationic structure and higher molecular weight of these antibiotics (12). Consequently, the CSF distribution of polymyxin after IV injection is relatively low, with a reported CSF-to-serum concentration ratio of 0.07 (3). To increase the concentration of polymyxin B in the CSF, the IVT route was conducted.

The IDSA guidelines state that if carbapenem resistance is suspected then a combination of IV and IVT polymyxin B is recommended, especially when systemic IV medication is not effective (4). Pan et al. (7) collected 61 cases with CSF culture of CRAB and found that the intrathecal/intracerebral polymyxin B group had significantly lower 28-day mortality and higher rates of microbiological clearance compared with the IV group. Therefore, the IVT+IV polymyxin B approach is suggested as the optimal treatment option for CNS infections with MDR/XDR Gram-negative bacteria. In the current study, IVT+IV polymyxin B was used to treat patients with CRAB or CRKP in CSF cultures, and all patients were successfully cured without severe adverse events or 28-day mortality.

Despite the success of IVT+IV polymyxin B treatment, the optimal dose/duration of IVT polymyxin B remains unclear. Current IDSA guidelines for IVT polymyxin B recommend a dose of 5 mg/day for 10–21 days (4). In the International Consensus Guidelines for the Optimal Use of the Polymyxins, the dose is 5 mg/day for IVT polymyxin B with a mean duration of 18 days (8). However, the quality of evidence is low as the guidelines have stated. Literature in the PubMed database with the keywords of intraventricular/intrathecal polymyxin B was reviewed (Table 6). Most of the identified papers/studies were case reports, including infants and pediatric patients. Chen et al. (16) reported a total of 28 patients with MDR/XDR Gram-negative bacilli intracranial infection after neurosurgery. IVT polymyxin B at a dose of 5 mg/day was used for 14.96 ± 4.28 days (range, 9–23 days) and the clinical cure rate was 82.1% (23/28). Chen et al. (17) recruited a total of 21 patients with MDR/XDR-AB-induced intracranial infection after neurosurgeries, all of which received IVT polymyxin B at a dose of 5 mg/day for 18.19 ± 12.36 days, and the clinical cure rate was 81.0% (17/21). In the present study, a daily IVT dose of 5 mg polymyxin B was used. The mean duration of administration was 14 ± 5.69 days (range, 6–20 days), the mean duration to achieve sterile CSF was 10.33 ± 3.67 days (range, 5–14 days), and the clinical cure rate was 100% (6/6). Based on existing evidence and data from the current study, a daily IVT dose of 5 mg polymyxin B is considered the optimal adult dose, and the treatment duration of IVT polymyxin B should be at least 10 days.

**Table 6:** Studies of CNS infections treated with intrathecal (IT) or intraventricular (IVT) polymyxin B^*^.

**Author/Year**	**No. cases**	**Pathogens (No.)**	**IT/IVT antibiotic and dosage**	**Duration of IT/IVT antibiotics (days)**	**Outcome**
Segal-Maurer et al. (13)	1 (Adult)	Ceftazidime-Resistant *K. pneumoniae*	Polymyxin B 5 mg/day	7	Cured
Piparsania et al. (14)	1 (32-week-old infant)	Carbapenem-resistant *A. baumannii*	Polymyxin B 4 mg/2 days	28	Cured
Guo et al. (15)	1 (Adult)	Carbapenem-resistant *A. baumannii*	Polymyxin B 5 mg/day for 10 days, then 2.5 mg/12 h	N/A	Cured
Pan et al. (7)	23 (Adults)	Carbapenem-resistant *A. baumannii*	Polymyxin B 5 mg/day	N/A	21/23 cured
Chen et al. (16)	28 (Adults)	Carbapenem-resistant *A. baumannii* (14); Carbapenem-resistant *K. pneumoniae* (9); Carbapenem-resistant *Pseudomonas aeruginosa* (3); Carbapenem-resistant *Enterobacter cloacae* (2)	Polymyxin B 5 mg/day	14.96 ± 4.28	23/28 cured
Zhong et al. (18)	1 (Adult)	Carbapenem-resistant *A. baumannii*	Polymyxin B 10 mg/day for 4 days, then 10 mg/2 days	19	Cured
Xing et al. (19)	1 (14-year-old adolescent)	Carbapenem-resistant *A. baumannii*	Polymyxin B 5 mg/day for 4 days, then 5 mg/2 days	9	Cured
Li et al. (20)	1 (Adult)	Carbapenem-resistant *A. baumannii*	Polymyxin B 5 mg/day + tigecycline 5 mg/day	16	Cured
Chen et al. (17)	21 (Adults)	Carbapenem-resistant *A. baumannii*	Polymyxin B 5 mg/day	18.19 ± 12.36	17/21 cured
Present study	6 (Adults)	Carbapenem-resistant *A. baumannii* (4); Carbapenem-resistant *K. pneumoniae* (2)	Polymyxin B 5 mg/day	14 ± 5.69	All cured

*CNS, central nervous system; IT, intrathecal; IVT, intraventricular; K. pneumoniae, Klebsiella pneumoniae; A. baumannii, Acinetobacter baumannii; N/A, Not applicable. Data of duration are presented as the mean ± standard deviation.*

*Database: PubMed; Keywords: [intraventricular polymyxin B(Title/Abstract)] OR [intrathecal polymyxin B(Title/Abstract)].

Regretfully, the concentration of polymyxin B in CSF, which might be able to guide the optimal dosage of polymyxin B, was not monitored in most studies. In a recent study, Ni et al. (21) administered colistin (also known as polymyxin E) at 100,000 units/24 h *via* IVT to 10 patients with CNS infections. Three days later, the concentration of colistin in the CSF was determined by selective ultra-performance liquid chromatography (UPLC) at 2, 4, 6, 8, 12, and 24 h after IVT colistin administration. The measured trough concentration was 1.12–8.33 μg/mL, which was above the minimum inhibitory concentration (MIC) of 0.5 μg/mL. Microbial cure was observed in all patients. However, information on the CSF pharmacokinetics of polymyxin B is lacking.

Polymyxin B has been reported to have side effects such as nephrotoxicity and skin hyperpigmentation. Using the definition from Kidney Disease Improving Global Outcomes (KDIGO) guidelines (22), no cases of AKI were observed in the current study. Furthermore, the incidence and severity of nephrotoxicity was reported to be rare and mild in other studies (7, 16). This suggests the dosage of combined administration of polymyxin B in the present study was comparatively safe for renal function. The kidneys are the primary sites of polymyxin elimination, thus the dosage must be carefully monitored (23), and it is therefore recommended to monitor the blood/CSF concentration of polymyxin B. In the current study, all patients were successfully cured without AKI, epilepsy or other adverse events of grade 3 or higher. Recent case reports have mentioned the adverse effect of skin hyperpigmentation following IV polymyxin B treatment (24–26), but this is usually mild and can gradually disappear after discontinuation of medication (27). In the present study, skin hyperpigmentation was observed in four patients (66.6%).

There are some limitations to the current study, including the small sample size, the retrospective nature of the study, the lack of control groups, and the absence of monitoring the concentration of polymyxin in the CSF or blood. These limitations must be considered when drawing conclusions from the study.

## Conclusion

In conclusion, IVT combined with IV polymyxin B is a safe and effective treatment for post-neurosurgical patients with intracranial infection caused by MDR/XDR Gram-negative bacteria. Furthermore, a daily IVT dose of 5 mg polymyxin B is considered the optimal adult dose. However, the small sample size and lack of a control group limit the reliability of such conclusions. Further large-scale randomized controlled trials are warranted to confirm the findings from this study.

## Data Availability Statement

The original contributions presented in the study are included in the article/supplementary material, further inquiries can be directed to the corresponding author.

## Ethics Statement

The studies involving human participants were reviewed and approved by Clinical Research Ethics Committee of the First Affiliated Hospital, College of Medicine, Zhejiang University. Written informed consent for participation was not required for this study in accordance with the national legislation and the institutional requirements.

## Author Contributions

HL, WY, and GW contributed to data collection. HL and WY performed the statistical analysis. HL wrote the first draft of the manuscript. WY, GW, and HC wrote sections of the manuscript. WY and HC reviewed the manuscript. All authors contributed to manuscript revision, read, and approved the submitted version.

## Conflict of Interest

The authors declare that the research was conducted in the absence of any commercial or financial relationships that could be construed as a potential conflict of interest.

## Publisher's Note

All claims expressed in this article are solely those of the authors and do not necessarily represent those of their affiliated organizations, or those of the publisher, the editors and the reviewers. Any product that may be evaluated in this article, or claim that may be made by its manufacturer, is not guaranteed or endorsed by the publisher.

## Footnotes

^1^ http://www.chinets.com
